# DNA Marker Transmission and Linkage Analysis in Populations Derived from a Sugarcane (*Saccharum* spp.) x *Erianthus arundinaceus* Hybrid

**DOI:** 10.1371/journal.pone.0128865

**Published:** 2015-06-08

**Authors:** Jian-wen Chen, Fang-ye Lao, Xi-wen Chen, Hai-hua Deng, Rui Liu, Hui-yi He, Cheng Fu, Yong-sheng Chen, Fu-ye Liu, Qi-wei Li, Phillip Jackson, Karen Aitken

**Affiliations:** 1 Guangdong Key Laboratory of Sugarcane Improvement and Biorefinery, Guangzhou Sugarcane Industry Research Institute, Guangzhou, China; 2 CSIRO Agriculture, Queensland Bioscience Precinct, Brisbane, Qld, Australia; South China Agricultural University, CHINA

## Abstract

Introgression of *Erianthus arundinaceus* has been the focus of several sugarcane breeding programs in the world, because the species has desirable traits such as high biomass production, vigour, ratooning ability and good resistance to environmental stresses and disease. In this study four genetic maps were constructed for two intergeneric populations. The first population (BC_1_) was generated from a cross between an *Erianthus/Saccharum* hybrid YC96-40 and a commercial sugarcane variety CP84-1198. The second population (BC_2_) was generated from a cross between YCE01-116, a progeny of the BC_1_ cross and NJ57-416, a commercial sugarcane cultivar. Markers across both populations were generated using 35 AFLP and 23 SSR primer pairs. A total of 756 and 728 polymorphic markers were scored in the BC_1_ and BC_2_ populations, respectively. In the BC_1_ population, a higher proportion of markers was derived from the *Erianthus* ancestor than those from the *Saccharum* ancestor Badila. In the BC_2_ population, both the number and proportion of markers derived from *Erianthus* were approximately half of those in the BC_1_ population. Linkage analysis led to the construction of 38, 57, 36 and 47 linkage groups (LGs) for YC96-40, CP84-1198, YCE01-116, and NJ57-416, encompassing 116, 174, 97 and 159 markers (including single dose, double dose and bi-parental markers), respectively. These LGs could be further placed into four, five, five and six homology groups (HGs), respectively, based on information from multi-allelic SSR markers and repulsion phase linkages detected between LGs. Analysis of repulsion phase linkage indicated that *Erianthus* behaved like a true autopolyploid.

## Introduction

Clones of the species *Erianthus arundinaceus* have a number of potentially useful agronomic traits such as high biomass production and vigour, tolerance to environmental stresses such as drought and waterlogging, and disease resistance [1–2]. *E*. *arundincaceus* belongs to the *Saccharum* complex, which consists of *Saccharum* and four other genera which form a potentially interbreeding group involved in the origin of sugarcane [3–4]. Sugarcane breeders have been interested in using *E*. *arundinaceus* in introgression programs largely because of a desire to incorporate some of the desirable traits into breeding parents and cultivars. A number of hybrids between *Saccharum officinarum* and *Erianthus* have been reported [5–9]. Although some sugarcane breeders claimed introgression of *Erianthus*, most used morphological characteristics for identification of hybrids, and these characteristics are unreliable. The first reports verifying successful production of fertile *Saccharum* x *E*. *arundinaceus* hybrids using DNA markers were published by Deng et al.[8] and Cai et al. [9]. This has opened up the possibility of introgression of favourable genome components from *E*. *arundinaceus* into sugarcane cultivars.

It has been suggested that DNA markers may be used to assist in introgression breeding [10–12]. This could occur through identifying markers linked to relatively favourable or unfavourable QTL derived from the exotic germplasm source being introgressed. These QTL may then be selected for or against during further crossing with commercial type parents. The work reported in this paper was part of a wider research to assess if and how DNA marker-assisted selection could be usefully applied in introgression of *Erianthus* in sugarcane breeding.

Sugarcane (*Saccharum* spp) is the most genetically complex crop species [13], and presents unusual challenges for application of DNA markers. Commercial sugarcane cultivars are derived from interspecific hybridisation between *S*. *officinarum* and *S*. *spontaneum*, and usually have between 100 and 120 chromosomes and with a map length believed to be around 17,000–18000 cM [14–16]. Most of the genome consists of *S*. *officinarum* chromosomes, with around 15–20% believed to be contributed by *S*. *spontaneum* [17]. *S*. *officinarum* (x = 10, 2n = 80) is an octoploid, while *S*. *spontaneum* (x = 8, 2n = 40–128) varies in ploidy level [18]. *E*. *arundinaceus* has been reported with chromosome numbers ranging from 2n = 20 to 60, with 2n = 40 and 60 karyotypes predominating [2].

Difficulties arise in DNA marker and QTL mapping in *Saccharum* spp. due to the high ploidy level and the large genome size. The best current maps published for sugarcane, based on about 2300 polymorphic markers, are believed to cover only about 60% of the genome [16]. Complexities arise in mapping in polyploids due to the large number of genotypes possible at each locus, and an inability to distinguish between genotypes with more than one copy (dosage level) of each allele. To overcome the difficulties due to polyploidy, mapping methods have been used which only use alleles present in either or both parents as a single copy (SD) [19]. These markers are identified initially through 1:1 segregation ratios. These markers can be mapped using similar methods as in diploids. In addition, co-dominant markers (eg. SSRs and RFLPs) can be used in identifying linkage groups belonging to the same homology groups. These approaches have been used to construct a number of maps for *Saccharum* species or sugarcane [14–15, 20–26]. More recently double dose (DD) markers were included along with SD markers in the development of a linkage map of *S*. *officinarum* [27]. No linkage maps involving *Erianthus* spp. have been reported to our knowledge.

In work leading to the current study, a hybrid clone produced from a cross between *S*. *officinarum* (2n = 80) and *E*. *arundinaceus* (2n = 60) was crossed to a commercial sugarcane cultivar to produce a progeny population potentially suitable for linkage and QTL mapping [9]. A clone from this population was then crossed to another commercial cultivar to produce a second population. Clones within either of these populations could be expected to be of value as parental material for commercial sugarcane breeding programs, through introduction of potentially valuable genome components derived from *E*. *arundinaceus*. Work by Piperidis et al [28] using GISH characterisation demonstrated that the BC_1_ population resulted from 2n + n transmission. The BC_1_ parent used in this study contained 117–122 chromosomes of which 91–94 were inherited from *Saccharum* and 25–30 from *Erianthus*. The three BC_2_ clones demonstrated n + n transmission from the BC_1_ and contained from 113–115 chromosomes of which 98–101 were inherited from *Saccharum* and 14–15 from *Erianthus*. In this study, we report on the transmission, segregation patterns, and linkage of DNA markers derived from *E*. *arundinaceus* and the *Saccharum* parents in these populations. This is intended to provide a basis for identifying markers linked to QTL in populations derived from *E*. *arundinaceus*, and to help determine if and how marker assisted selection could be used to assist in the future introgression of components of the *E*. *arundinaceus* genome in sugarcane breeding programs.

## Materials and Methods

### Plant material

**Fig 1:**
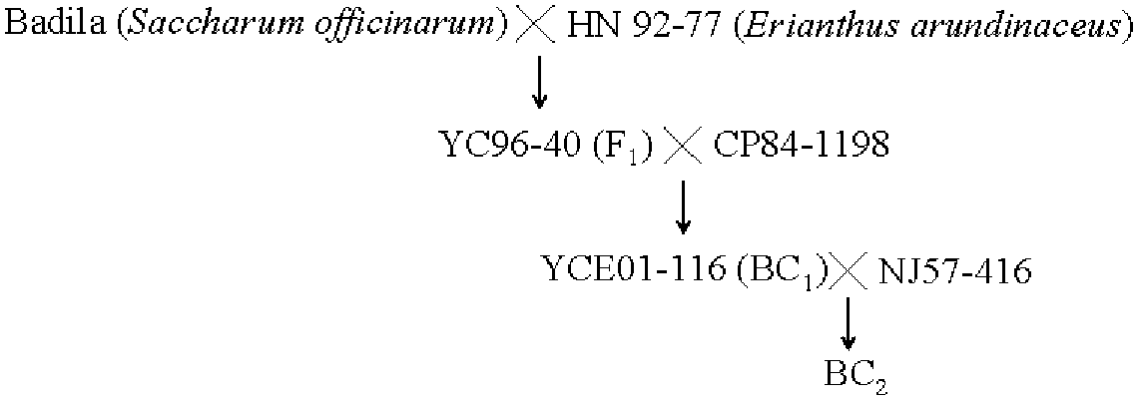
Pedigree tree of BC_1_ and BC_2_ populations used in this study.

Two populations, termed BC_1_ and BC_2_ (referring to first and second backcrosses to commercial type parents after the original inter-generic cross) were derived from crosses YC96-40 × CP84-1198 and YCE01-116 × NJ57-416. These consisted of 173 and 168 clones, respectively, and were used as mapping populations in this study. The clone of YC96-40 is from the intergeneric cross Badila (*S*. *officinarum*) × HN92-77 (*Erianthus arundinaceus*, collected from Yacheng, Sanya, Hainan, China.). YCE01-116 is one of the progeny of the BC_1_ population. NJ57-416 is a Chinese sugarcane cultivar, and was bred in mainland China. Their pedigree tree for these populations is shown in Fig 1.

All crosses were made by The Guangzhou Sugarcane Industry Research Institute (GSIRI) at the Hainan Sugarcane Breeding Station, Hainan province, China. The two populations, along with the parents, were sampled from the germplasm collection maintained by the Hainan Sugarcane Breeding Station. All progeny were tested with *Erianthus*-specific markers [29], SSRs [9], and AFLPs (data not shown) prior to linkage analysis to confirm that they were genuine hybrids.

### DNA extraction

Fresh leaf tissue was collected from the two populations in the field, placed on ice and eventually stored in a refrigerator until DNA extraction. For DNA extraction, samples were frozen and then ground to powder in liquid nitrogen. Genomic DNA was extracted using the CTAB method described by Hoisngton [30]. The quality of the DNA was checked by running the samples on the 0.8% agarose gel. Then the DNA was stored at -20°C until used.

### SSR analysis

SSR primers were obtained from CSIRO Plant Industry (now CSIRO Agriculture), Australia, and were synthesized by Shanghai Sangon Biotechnology Co., Ltd. (Shanghai, China). These were screened against the parents of the two populations to determine polymorphism levels. A total of 23 primer pairs were selected and scored across the BC_1_ and BC_2_ populations using the method described by Aitken et al. [15] with some modifications. The PCR reactions for the SSRs were carried out in a total volume of 20 μl containing 5 μl of 1/20 dilution of DNA sample (25–30 ng DNA), 2.4 μl of 25 mM MgCl_2_, 1.6 μl of 2.5 mM dNTPs, 2.0 μl of 10 × Buffer, 0.16 μl each of 30 μM/μl forward and reverse primers, 0.1 μl of 5 U/μl *Taq*, and 8.58 μl of distilled water. PCR amplification reactions were performed on the Eppendorf Mastercycler Gradient (Germany) or the GeneAmp PCR System 9700 (PE Applied Biosystems, Foster City, CA, USA) under the program of 94°C for 3 min, 35 cycles of (94°Cfor 1 min, annealing for 2 min, and 72°C for 1 min), with a final extension at 72°C for 5 min, and holding at 4°C. The annealing temperature varied from 50°C to 56°C depending on each SSR primer pair. The amplified products were mixed with an equal volume of loading dye, denatured at 95°C for 5 min, and 5 μl samples run on a denaturing 5% polyacrylamide (20:1) at 90W for 2 hours. The gels were visualized using the method of silver staining described by Sanguinetti et al. [31] and modified according to Xu et al. [32].

### AFLP

A total of 35 AFLP primer pairs were used to fingerprint all individuals of the BC_1_ and BC_2_ populations. Four of them were performed according to the protocol of Aitken et al. [15]. The others were conducted following the method described by Aitken et al. [15] with modifications. Genomic DNA (300–400 ng) of each genotype was double digested with *Eco*RI and *Mse*I restriction enzymes and ligated to the adapters specific for the *Eco*RI and *Mse*I restriction sites. A pre-selective amplification was carried out with *Eco*RI +A and *Mse*I +C primers. The resultant PCR products were then diluted 1:20 with pure distilled water and used as template for the selective amplifications. The selective amplifications were performed with three selective nucleotides in a final volume of 20 μl. PCR products were separated as described for the SSR markers and the markers visualised by silver staining the gels.

### Marker scoring and analysis

All segregation bands that were distinct and unambiguous for most clones were scored (1 for present, 0 for absent and—for missing). Each marker system identified both monomorphic and polymorphic markers. Each marker was tested against expected segregation ratio using a χ^2^ test for SD, DD and bi-parental single dose (segregation ration of 3:1). SD markers are present only once in the genome, either in a 1:1 ratio (markers present once in one parental genome) or in a 3:1 ratio (marker present in both parents segregating as SD markers called bi-parental single dose). DD markers are present twice in one parental genome, either in an 11:3 ratio (for x = 8) or in a 7:2 ratio (for x = 10) [24]. AFLP markers were denoted by three respective selective nucleotides of *Eco*RI-*Mse*I primer pairs, followed by number of polymorphic bands in descending molecular-weight order. SSR markers were identified by the name from the Sugarcane Microsatellite Consortium collection, followed by number of polymorphic bands in descending molecular-weight order. In the BC_1_ population, if the marker was derived from the parent “YC96-40” and originally from the ancestor parent “*E*. *arundinaceus* viz. HN92-77”, it was named with a trailing E; if it was derived from the ancestor “Badila”, then it was named with a trailing S; if both ancestors presented the band, then it was named with a trailing B; if the band was not clear in either ancestral parent it was named with a trailing U. In the BC_2_ population, if the marker was derived from the parent “YCE01-116”, it was also labelled with a trailing E, S, B or U, following the same criteria.

### Linkage map construction

All linkage maps were constructed following methods used by Aitken et al. [27] with slight modification. Initially single dose markers were used to determine linkage relationships in coupling phase linkage using JoinMap 4 [33]. Grouping of markers and map construction was carried out at a LOD of 5 with map distances from the recombination fractions derived using the Kosambi function. Coupling linkage among dominant single dose markers should result in 2n linkage groups [25]. This fixed order single dose map was used as a framework map to assign double dose and bi-parental single dose markers, and lastly repulsion phase markers were added. To do this, recombination fraction (r) and LOD scores for all combinations of marker were calculated as in Aitken et al.[27]. Such pairwise estimates are suitable for input into the JoinMap 4. The set of pairwise estimates of r and LOD under the octosomic model were used to locate duplex and double simplex markers on the framework map.

### Homologous groups

The LGs were formed into homologous groups by using the SSR loci that had been mapped in Aitken et al.[15]. Groups were also formed using the markers that were polymorphic in both crosses and markers that were scored across the two generation.

## Results

### BC_1_ population

**Fig 2:**
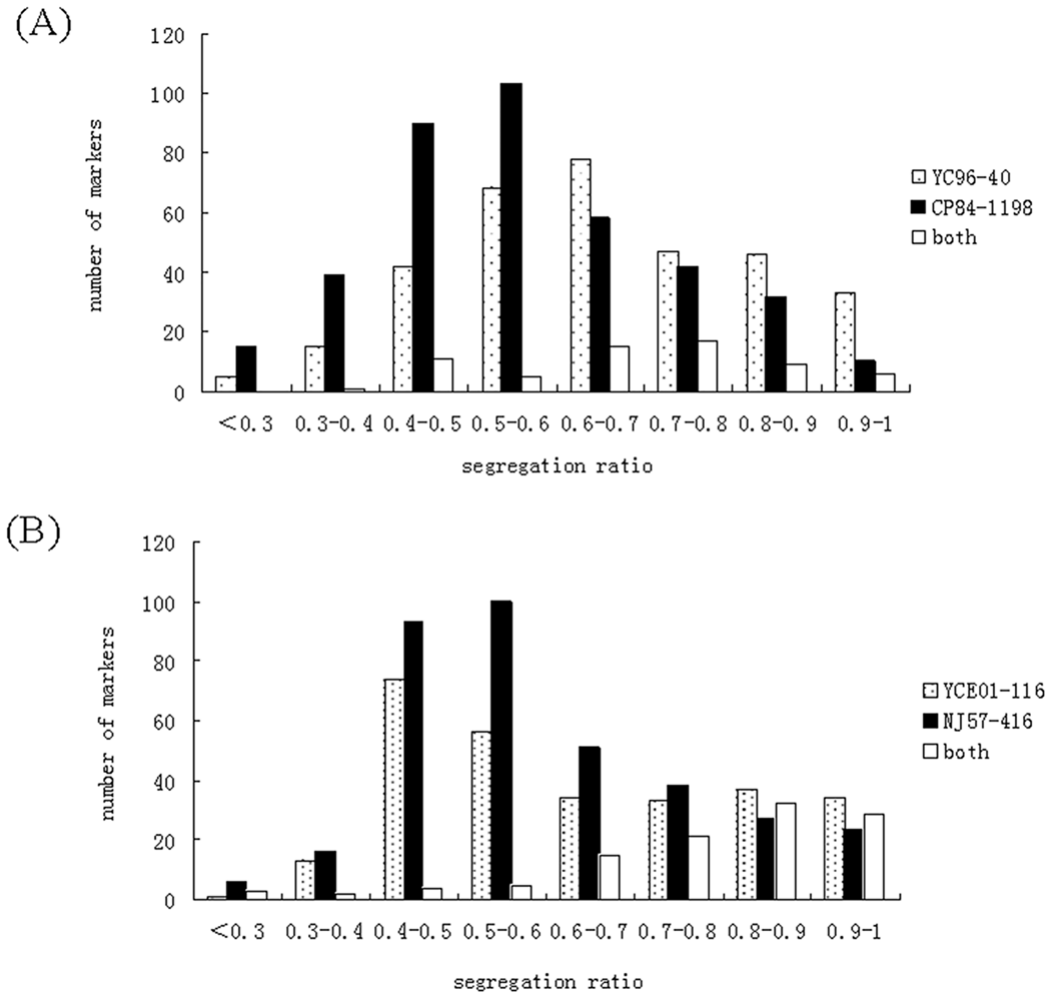
Frequency distribution of the markers (derived from AFLP and SSR primer combinations) in the BC_1_(A) and BC_2_(B) generations.

A total of 756 unambiguous polymorphic markers were detected after genotyping 173 BC_1_ progeny from the cross YC96-40 x CP84-1198 with 35 AFLP and 23 SSR primer pairs. The distribution of segregation ratios of these are shown in Fig 2(A). The 35 AFLP primer combinations generated 648 polymorphic markers in this population. Each AFLP primer combination detected 6 to 38 polymorphic markers, of which 3 to 19 were simplex markers. The 23 SSRs detected a total of 108 markers, with each SSR primer pair detecting 2 to 12 markers, of which 2 to 12 were simplex markers. In total, 312 markers were scored that were present in YC96-40 and absent in CP84-1198, of which 119 and 93 were SD markers and duplex markers, respectively (Table 1). There were a total of 381 markers present in CP84-1198 and absent in YC96-40, with the SD markers (217) comprising a higher proportion than the duplex markers (81), in contrast with markers derived from the YC96-40 (Table 1). An additional 59 bands were present in both parents of the BC_1_ population.

**Table 1:** Number of markers derived from different ancestors in each of the two populations.

Source/s of markers		Type of marker			
		Non polymorphic	Single dose	Double dose	Higher dosage
BC_1_ population^a^					
Present in YC96-40 but not in CP84-1198	Only in Badila	6	34	21	12
	Only in HN92-77	10	85	72	72
	In both Badila and HN92-77	1	6	9	5
Present in CP84-1198 but not in YC96-40		0	217	81	83
In both parents	Only in *Saccharum* ^c^	133	20	19	
	In both *Saccharum* and *Erianthus*	219	11	9	
BC_2_ population^b^					
Present in YCE01-116 but not in NJ57-416	Only in *Saccharum*	130	81	19	20
	Only in HN92-77		49	48	41
	In both *Saccharum* and HN92-77	65	8	6	4
Present in NJ57-416 but not in YCE01-116		5	211	65	77
In both parents	Only in *Saccharum*	135	34	44	
	In both *Saccharum* and HN92-77	68	6	15	

Markers are separated into different groups (non polymorphic, and single-, double- and higher dosage markers, based on segregation ratios).

^a^ An additional 28 markers were scored in the BC_1_ population, but were not scored clearly in either parent.

^b^ An additional 19 markers were scored in the BC_2_ population, but were not scored clearly in either parent.

^c^ “*Saccharum*” means from Badila or CP84-1198 or NJ57-416.

In considering markers present in only YC96-40 but not in CP84-1198, a higher proportion were derived from the *Erianthus* ancestor than from the *Saccharum* ancestor (Badila). A high proportion of markers present in both parents were non polymorphic.

Markers derived from the *Erianthus* ancestor also displayed a different distribution of segregation ratios than from the *Saccharum* parents (Table 1). There was a lower proportion of markers derived only from the *Erianthus* ancestor which segregated as single dose markers (approximately 35%) compared with for the *Saccharum* parents (approximately 50%). Conversely a higher proportion of higher dosage markers was present in the *Erianthus* ancestor.

### BC_2_ population

In total, 728 unambiguous polymorphic markers were obtained after genotyping 168 BC_2_ progeny clones from the cross YCE01-116 x NJ57-416 using the same AFLP and SSR primer pairs as used for the BC_1_ progeny clones. The distribution of segregation ratios of markers derived from each parent is shown in Fig 2(B). The 35 AFLP primer combinations generated 605 out of these 728 markers, with each AFLP primer combination detecting between 5 to 42 polymorphic markers, of which 1 to 24 were simplex markers. The 23 SSR primer pairs detected a total of 123 markers, with each pair detecting between 2 to 13 markers, of which 1 to 10 were simplex. In total, 276 markers were specific to YCE01-116 of which 138 and 73 were SD markers and duplex markers, respectively (Table 1). Altogether 353 markers were found to be NJ57-416-specific and absent in YCE01-116, and the number of SD markers (211) was 3.24 times the number of duplex markers (65) (Table 1). Ninety-nine markers were present in both parents.

Further analysis of the ancestral sources of the markers present in the *Erianthus* derived BC_1_ parent YCE01-116 revealed some differences with the *Erianthus* derived F_1_ clone YC96-40. For the SD markers inherited from YCE01-116, 35.5% (49) of them were derived only from *Erianthus*, and 58.7% (81) from *Saccharum*. This contrasts with YC96-40 where the ratio between *Erianthus* and *Saccharum* derived SD markers was the opposite. For double dose and higher dosage markers both the number and proportion (relative to *Saccharum*) of markers derived from *Erianthus* in the BC_2_ progeny population was approximately half of that in the BC_1_ population.

Comparing the markers inherited from *Erianthus* in the parents of YC96-40 and YCE01-116, the marker number and proportion in the latter was approximately half of that in the former, which is consistent with the *Erianthus* chromosome transmission of from 2n (in BC_1_) to n (in BC_2_).

### Linkage mapping

In both generations there were more markers included in the sugarcane (*Saccharum*) genetic maps than the *Erianthus*/sugarcane hybrid maps which is consistent with the higher number of polymorphic markers scored for the sugarcane parents (Table 1). For both parents derived from *Erianthus* there was a greater proportion of double dose markers (compared with SD markers) included in the linkage maps than for the *Saccharum* parents, with YC96-40 and YCE01-116 having 30% and 24% respectively of the markers in their maps being double dose markers, compared with 18% and 14% for the *Saccharum* parents in the same populations respectively (Table 2). The *Saccharum* parent maps had higher numbers of linkage groups than the parents derived from *Erianthus* (Table 3). This is a reflection of the higher overall number of markers identified in these clones, contributed by larger numbers of SD markers. The *Saccharum* parents also had a higher number of LGs assigned to HGs and this was due to a larger number of polymorphic SSR markers identified in these parents. Repulsion phase markers were added as a last addition to these linkage maps. The number of repulsion phase markers included varied between linkage maps with the lowest number 4 (5%) detected in YC96-40 and the highest in NJ57-416 (16%).

**Table 2:** Number of markers in the BC_1_ and the BC_2_ generations included in the linkage maps.

Population/parent	Number of single dose makers	Number of double dose markers	Number of bi-parental markers	Total number of markers
**BC_1_**				
**YC96-40**	79	31 (30%)	6	116
**CP84-1198**	137	31 (18%)	6	174
**BC_2_**				
**YCE01-116**	67	23 (24%)	7	97
**NJ57-416**	129	23 (14%)	7	159

Markers are separated into different groups (non polymorphic, and single-, double- and higher dosage markers, based on segregation ratios)

**Table 3:** Number of linkage groups (LGs) assigned to homology groups (HG’s) and origin of inheritance of these for each linkage map.

Population/parent	No. LGs	Inheritance			No. of LGs in HGs	No. of Unassigned LGs
		*Erianthus*	*Saccharum*	Recom.		
**BC_1_**						
**YC96-40**	38	26	12	-	12	26
**CP84-1198**	57	-	-	-	31	26
**Bi-parental**	1					
**BC_2_**						
**YCE01-116**	36	17	15	4	15	21
**NJ57-416**	47	-	-	-	24	23
**Bi-parental**	3					

### BC_1_ Linkage maps

The YC96-40 linkage map consisted of 38 linkage groups each with 2 to ten markers. These ranged in length from 0.1 cM to 108.7 cM. The total map length was 1209.7 cM which gave an average distance between markers of 10.4 cM (Table 4). The CP84-1198 linkage map contained 57 linkage groups each with 2 to 14 markers with a length from 5.5 cM to 196.8 cM. The total map length was 2283.5 cM with an average distance of 13.6 cM between markers (Table 4). CP84-1198 is a hybrid between *S*. *officinarum* and *S*. *spontaneum* and the longer chromosomes are probably those inherited form the *S*. *spontaneum* part of the genome which is more polymorphic [14].

**Table 4:** Total Map lengths (cM) for each linkage map and proportion of total linkage map length (cM) contributed from each genus.

Population/parent	Total Map length (cM)	*Erianthus* Map length	*Saccharum* Map length	RecombinantMap Length
**BC_1_**				
**YC96-40**	1209.7	887.6	322.1	
**CP84-1198**	2283.5		2283.5	
**Bi-parental**	103.1			
**BC_2_**				
**YCE01-116**	973.9	484.8	329.2	159.9
**NJ57-416**	1955.5		1955.5	
**Bi-parental**	96.5			

### BC_2_ Linkage maps

**Fig 3:**
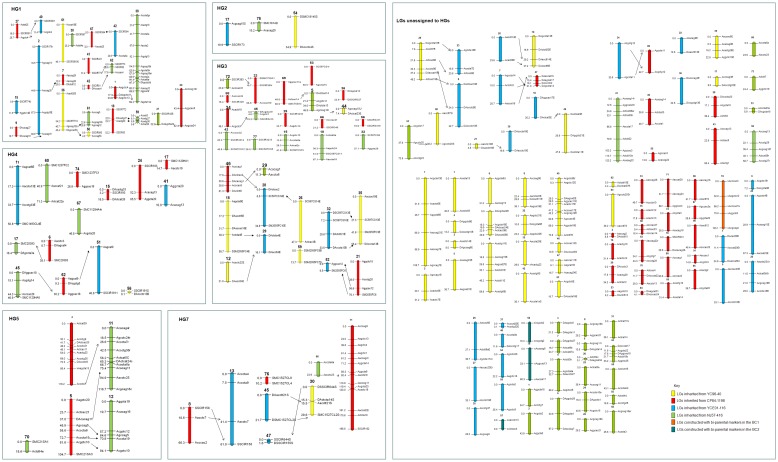
A combined linkage map of the four parents in this study. For YC96-40 and YCE01-116 markers inherited from *Erianthus* have an E at the end of the marker name, markers inherited from the *Saccharum* parent if they can be identified have an S at the end of the marker name, if the marker origin is unknown then a U is added to the end of the marker name. Double dose markers have a D at the start of the marker name and bi-parental markers have an X. Arrows indicate the same marker inherited from one generation to the next or identified in both *Saccharum* parents.

The YCE01-116 linkage map consisted of 36 LGs, 17 inherited from *Erianthus*, 15 from *Saccharum* and 4 that appear to be recombinant (Table 3 and Fig 3). The LGs ranged in length from 3.1 cM to 88.9 cM. The total map length was 973.9 cM with and average distance between markers of 11 cM. Some of the markers could be traced back to the *S*. *officinarum* parent Badila. LG 45 and 47 in HG7 appear to be *S*. *officinarum* in origin and the rest of the *Saccharum* inherited LGs have markers that are from the CP84-1198. The NJ57-416 linkage map contained 47 LGs each with 2 to 14 markers ranging in length from 0.1 cM to 194.2 cM. The total map length was 1955.5 cM which gave an average distance of 12.3 cM between markers (Table 4). Again this map contained some longer LGs which are probably from the *S*. *spontaneum* part of the genome.

### Transmission of LG from the F_1_ to the BC_1_

All the markers were scored consistently across the generations so that transmission of the LGs could be determined. Eight linkage groups could be followed that were inherited from the *Erianthus* parent YC96-40 to YCE01-116 (Fig 3). Four of these linkage groups were in HG3, one in HG7 and three further were unassigned to HGs.

## Discussion

Four linkage maps were constructed from the two intergeneric populations, YC96-40 and YCE01-116 (two hybrids of *Erianthus*/sugarcane) and two commercial cultivars (namely CP84-1198 and NJ57-416), using 35 AFLP and 23 SSR primer pairs. This is the first report on linkage maps constructed using intergeneric populations involving introgression of *E*. *arundinaceus* into sugarcane. The maps of YC96-40, CP84-1198, YCE01-116 and NJ57-416, consisted of 116 (45.0% of the total number of polymorphic markers derived from this parent), 174 (52.9%), 97 (38.6%) and 159 (50.3%) markers, respectively, leaving many SD and DD markers unlinked. Many LGs had only two to three markers, and the map lengths (1209.7 cM, 2283.5 cM, 973.9 cM and 1955.5 cM, respectively) are much shorter than seen in other linkage map research in sugarcane. The reasons attributed to this would include: 1) silver staining, was used for visualizing the gels which is not as sensitive as using radio labelled methods of detection so less markers could be scored; and 2) a stringent LOD value of 5 was used while developing the linkage map [34] to prevent spurious associations and introduction of errors into the linkage maps.

In the present research, the LGs of YC96-40, CP84-1198, YCE01-116 and NJ57-416 could be placed in 4, 5, 5 and 6 homology groups (HGs), respectively, using the same methods described by Aitken et al. [27] (Fig 3). In total 55% of LGs could not be placed in a HG (Fig 3, Table 3). The main reason for this is the lower level of polymorphism detected in these populations compared to other *Saccharum* mapping populations [27]. 2n transmission from the F_1_ to the BC_1_ as reported by Piperidis et al. [28] would also reduce the level of polymorphism and increase the number of double dose markers as seen in the data. The LGs were assembled into HGs using previous information from other *Saccharum* mapping populations and it is possible that duplication of some SSR locus within the basic chromosome set may lead to mis-assembly of sets of homologous LGs into the same HG [35]. It is probable that the basic chromosome number for *Erianthus* is *x* = 10 but it has not as yet been confirmed and this could also lead to mis-assembly of HGs. Repulsion phase markers were used to improve map coverage and determine the probability of preferential pairing. The sugarcane cultivars CP84-1198 and NJ57-416 had 4.7% and 11.8% respectively of markers linked in repulsion. The F_1_ had only 2.5% markers linked in repulsion indicating that *Erianthus* had little or no preferential pairing and behaved like a true autopolyploid. The BC_1_ clone YCE01-116 had 10% markers linked in repulsion, similar to the cultivars, indicating that more preferential pairing was occurring probably due to the increase in *S*. *spontaneum* chromosomes which could preferentially pair. This preferential pairing of LGs inherited from *S*. *spontaneum* has been seen in other *Saccharum* linkage maps [14–15].

### YC96-40 linkage map

The linkage map contained 26 LGs inherited from the *Erianthus* parent and 12 from the *Saccharum* parent. GISH analysis of this F_1_ clone showed that it contains 70 chromosomes, 40 inherited from *Saccharum* and 30 from *Erianthus* [28]. While the linkage map appears to include a higher proportion of the present *Erianthus* chromosomes than for the *Saccharum* chromosomes, the length of the map indicates that the genome coverage is low. Of the double dose markers in the linkage map 42% were inherited from the *Erianthus* parent as opposed to 6% from the *Saccharum* parent. The higher proportion of higher dosage markers inherited from the *Erianthus* parent is consistent with low polymorphism levels seen in *Erianthus* germplasm compared to *Saccharum* [36].

### Inheritance of *Erianthus* linkage groups

The F_1_ parent YC96-40 inherited 26 linkage groups from *Erianthus* and 12 from *Saccharum*. As this map is of the F_1_ we would expect to inherit half the chromosomes of the *Saccharum* parent Badila which has 80 chromosomes and half of the 60 chromosomes of HN92-77 the *Erianthus* parent. The lower number of *Saccharum* linkage groups is probably due to lower numbers of single and double dose markers detected.

### YCE01-116 linkage map

The linkage map contained 17 LGs inherited from the *Erianthus* parent and 15 from the *Saccharum* parent and four recombinant LG (LG25) (Fig 3). Three out of the four linkage groups were linked in repulsion indicating possible pairing between a *Saccharum* and *Erianthus* chromosome. GISH analysis of this BC_1_ clone showed it contains 117–122 chromosomes, 91–94 inherited from *Saccharum* and 25–30 from *Erianthus*, no recombinant chromosome were identified [28] although only one individual was tested. This work demonstrated that 2n transmission from the F_1_ parent had occurred from the F_1_ to the BC_1_ generation this would account for the low polymorphism level in this generation and the larger number of double dose markers. Of these double dose markers included in this linkage map 79% were inherited from *Erianthus* and 21% from the *Saccharum* parent.

Although the linkage maps reported here are the first to be reported for intergeneric populations involving *Erianthus* and *Saccharum*, there is low genome coverage which is a common problem in genetic maps of sugarcane. This is due to the complex inheritance patterns of high level polyploids and the large chromosomes numbers. Sugarcane cultivars have from 100–120 chromosomes [17–18] so even maps with over 1000 markers still have only 60% genome coverage [15]. The addition of *Erianthus* in these intergeneric populations has complicated linkage map production by reducing the amount of polymorphism due to the 2n transmission and the inherently lower polymorphism present in *Erianthus* genomes.

These populations have verified that it is now possible to incorporate traits from *Erianthus* into sugarcane cultivars. The indication of pairing between the *Saccharum* and *Erianthus* chromosome demonstrates the possibility of recombination between these two genomes. This means that it may be possible to incorporate genes conferring traits like drought tolerance and disease resistance which may be derived from *Erianthus* without incorporating unwanted linked genome components from *Erianthus* that might be detrimental to sugarcane cultivars.

In introgression breeding in sugarcane the general objective is usually to incorporate desirable genome components from the wild cane into commercially elite parents. This is done through successive backcrossing of progeny to elite commercial type parents, to regain desirable agronomic traits while at the same time hoping to retain the desirable components. However, each successive backcross involves loss of genome from the wild cane, as observed in this study, and therefore a high risk of losing the most beneficial parts of the wild genome. DNA markers may play a valuable role in introgression breeding if DNA marker data such as that used in this study is used in QTL mapping in advanced backcrosses to identify beneficial parts of the wild cane genome. If this can be done, deliberate selection of progeny retaining markers linked to those beneficial genome components may be selected in each successive generation.

Further work is needed to incorporate more markers into these linkage maps to allow identification of association between markers and traits of interest. These populations could be further crossed to *Saccharum* to reduce the number of *Erianthus* chromosomes in the *Saccharum* background and encourage recombination between *Saccharum* and *Erianthus* chromosomes. These populations have made available the *Erianthus* genome for trait discovery by sugarcane breeders and QTL mapping, combining trait data and additional markers, may be useful for assessing breeding value of different sections of the *Erianthus* genome for sugarcane improvement.
